# Cortical Gray Matter in Attention-Deficit/Hyperactivity Disorder: A Structural Magnetic Resonance Imaging Study

**DOI:** 10.1016/j.jaac.2009.11.008

**Published:** 2010-03

**Authors:** Martin J. Batty, Elizabeth B. Liddle, Alain Pitiot, Roberto Toro, Madeleine J. Groom, Gaia Scerif, Mario Liotti, Peter F. Liddle, Tomáš Paus, Chris Hollis

**Affiliations:** a University of Nottingham; b Pasteur Institute, Paris, France; c University of Oxford; d Simon Fraser University, Canada; e Montreal Neurological Institute, Montreal, Quebec, Canada

**Keywords:** Attention-deficit/hyperactivity disorder, MRI, Cortical thickness, Inferior frontal gyrus, Gray matter

## Abstract

**Objective:**

Previous studies have shown smaller brain volume and less gray matter in children with attention-deficit/hyperactivity disorder (ADHD). Relatively few morphological studies have examined structures thought to subserve inhibitory control, one of the diagnostic features of ADHD. We examined one such region, the pars opercularis, predicting a thinner cortex of the inferior frontal gyrus (IFG) in children with ADHD.

**Method:**

Structural images were obtained from 49 children (24 control; 25 ADHD combined subtype) aged 9 though 15 years. Images were processed using a volumetric pipeline to provide a fully automated estimate of regional volumes of gray and white matter. A further analysis using FreeSurfer provided measures of cortical thickness for each lobe, and for 13 regions in the frontal lobe.

**Results:**

Relative to controls, children with ADHD had smaller whole brain volume and lower gray matter, but not white matter, volumes in all lobes. An analysis of frontal regions showed a significant interaction of group by region. Planned contrasts showed bilateral thinner cortex in the pars opercularis in children with ADHD.

**Conclusions:**

Children with ADHD showed both diffuse and regional gray matter abnormalities. Consistent with its putative role in response inhibition, the cortex of the pars opercularis was thinner in children with ADHD who, as expected, had significantly poorer inhibitory performance on a Go/No-go task. These differences held for both hemispheres raising the possibility that a developmental abnormality of IFG might drive development of inhibition difficulties.

Attention-deficit/hyperactivity disorder (ADHD) is a common neurodevelopmental disorder affecting between 3% and 5% of children and young persons. It is characterized by pervasive and developmentally inappropriate levels of inattention, impulsivity, and hyperactivity and wide-ranging behavioral and cognitive impairments, including deficits in working memory, inhibitory control, and altered motivational style.^1^ Disinhibited behavior is one of the diagnostic criteria for ADHD combined subtype, and children with ADHD typically perform more poorly than controls on tasks that require inhibition or suppression of a pre-potent response.^2^ This can be readily observed using paradigms such as the Go/No-go and Stop-Signal task in which subjects are required to make speeded responses to frequently presented targets while withholding responses to rarer “No-go” or “Stop” trials. Both tasks are thought to tap inhibitory behavioral control, a process generally considered to be supported by fronto-cortical regions.^3^

The frontal lobe is an important focus of research in ADHD for a number of reasons. Converging evidence implicates dopaminergic dysfunction of fronto-striatal circuits in the pathogenesis of the disorder.^4^ Studies using functional magnetic resonance imaging (fMRI) have shown differential task-related response in frontal regions in both children^4^ and adults^5^ with ADHD, relative to matched controls, in paradigms requiring response inhibition. Further evidence of the importance of the frontal lobes in ADHD comes from MR-based anatomical studies. Although interpretation of the anatomical findings is complicated by differences in methodology, clinical subtype and comorbidities (reviewed by Krain et al.^6^), children with ADHD appear to have smaller volumes of prefrontal^7^ and striatal^4,8^ gray matter (GM) as compared with typically developing children. Moreover, the presence of a relationship between performance in “inhibitory” tasks and fronto-striatal volume^4^ suggests a close association between the structural development of the fronto-cortical systems and cognitive functions implicated in ADHD symptoms.

Several fronto-cortical regions, including the ventrolateral prefrontal, dorsolateral prefrontal, and anterior cingulate cortex (ACC), are important in attention. In particular, several studies have highlighted the role of the ventrolateral regions of the inferior frontal gyrus (IFG)—particularly the right IFG—as being crucial for inhibiting behavioral responses.^9,10^ The IFG comprises three adjacent regions: pars opercularis Brodmann area (BA 44), pars triangularis (BA 45), and pars orbitalis (BA 47/12). Using a group of patients with lesions confined to the right frontal lobe, Aron et al.^9^ investigated the putative role of the IFG in response inhibition. They hypothesized that damage to IFG, but not other structures, would correlate with performance on a Stop-signal task. As predicted, there was a significant positive correlation between the extent of IFG damage and Stop Signal Reaction Time (SSRT), an inferred measure of the time taken to countermand a motor response. Closer examination of specific regions within IFG revealed that the damage of the pars opercularis, but not the pars triangularis, was a significant predictor of SSRT.

Further support for the importance of the pars opercularis in inhibitory control is provided by a recent repetitive transcranial magnetic stimulation (rTMS) study^10^ in which rTMS was applied over three regions of the right hemisphere: IFG (pars opercularis), middle frontal gyrus (MFG) and angular gyrus (AG). Participants completed two blocks of a Stop-signal task, each block preceded by a 15-minute period of 1Hz rTMS. Inhibitory performance was impaired only after applying rTMS over the IFG. Finally, as one might expect of a disorder in which inhibitory performance is compromised, structural^11,12^ and functional^4,13^ abnormalities in IFG have been noted in children with ADHD.

Although these findings highlight the significance of the IFG in inhibitory control, a number of other regions appear to be important for this function, including pre–supplementary motor area (preSMA) and right dorsolateral prefrontal and inferior parietal areas, with activation in the latter two regions being particularly pronounced when working memory load is high.^14^ In addition, fMRI signal in frontal (anterior cingulate), striatal and medial temporal regions has been shown to correlate with Stop Signal Reaction Time (SSRT) in children with ADHD.^15^ In support of this more “global” model of inhibitory deficits in ADHD, reductions in total brain volume^7,16^ and gray matter (GM) volume^17^ are among the most consistent structural findings in ADHD. In addition, thinner cerebral cortex in children with ADHD, as compared with typically developing children, has been found both globally and locally, most notably in frontal and central regions.^18^ Overall, the heterogeneity of findings in ADHD suggests that the disorder is likely to be characterized by widespread changes in cortical GM.^12,19^ Because the attention system of the human brain is thought to involve a number of distinct but interconnected regions,^20^ and because of the frequent occurrence of comorbid disorders in ADHD^21^ in which the neural bases are largely unknown, an examination of global changes in brain structure should prove useful.

Here, we used MRI to obtain a number of global and regional measures of brain structure in a clinical group of children and adolescents with ADHD characterized by clinical impulsivity/hyperactivity and inattention (DSM-IV combined subtype) and in healthy controls. In accordance with previous studies and the heterogeneous nature of the disorder, we predicted smaller brain volume and global reductions in GM in participants with ADHD. In addition, based on the putative role of the IFG in inhibitory control^9,10,22^ and the occurrence of inhibitory deficits in the disorder,^23^ we predicted lower cortical thickness in the right pars opercularis in participants with ADHD.

## Method

### Participants

Ethical approval was granted by the local Research Ethics Committee and Research and Development Departments of the Nottinghamshire Healthcare and Lincolnshire Partnership NHS Trusts. After complete description of the study, written informed consent and verbal assent was obtained from parents and children, respectively. Thirty children with ADHD and 33 controls taking part in an fMRI/EEG study (reported elsewhere^24^) underwent MRI scanning. Eleven children (five subjects with ADHD and six controls) were excluded because of excessive movement during scanning and three further controls were removed from the FreeSurfer analysis due to poor registration of their scans. Thus, data from 52 subjects were included in the volumetric analysis and 49 subjects were included in the cortical thickness analysis; 25 ADHD (24 male; mean age, 12.48 years; SD, 1.86) and 24 controls (23 male; mean age, 12.82; SD, 1.69). Details of clinical and demographic data are summarized in Table 1.

#### ADHD Group

Children and adolescents with a clinical diagnosis of ADHD were recruited from child psychiatry and community paediatric clinics. An initial telephone call to parents screened for broad inclusion criteria (9 through 15 years, right handed, currently taking and responsive to stimulant medication) and exclusion criteria (presence or history of psychosis, epilepsy or Tourette syndrome, diagnosis of moderate or severe learning disability, current use of nonstimulant or other psychotropic medication except melatonin). Eligible participants were invited to attend an assessment session in which we administered the Development and Well Being Assessment [DAWBA]^25^ and a battery of questionnaires were administered: Social Communication Questionnaire [SCQ],^26^ Strengths and Difficulties Questionnaire [SDQ],^27^ and Conners long form.^28^ Permission to access the child's medical records and contact their school was obtained, and teacher versions of the DAWBA, SDQ, and Conners were also completed for each child.

ADHD diagnosis was confirmed or overturned following a clinical consensus diagnostic meeting involving CH and another experienced child and adolescent psychiatrist. This included a full review of the child's medical history, parent and teacher DAWBA transcripts (including computer generated predictions) and questionnaires. Only right-handed participants with a DSM-IV diagnosis of ADHD combined subtype and an established positive response to stimulant medication (assessed using symptom severity and clinical interview ratings on and off medication) were included. Diagnosed or suspected comorbidities were investigated where necessary, and any participants with psychosis, bipolar disorder, major depression, Tourette syndrome, Autistic Disorder/Asperger's Disorder, major head trauma or epilepsy were excluded. Participants with comorbid oppositional defiant disorder (ODD), conduct disorder (CD), and anxiety disorder were included. A separate session assessed intelligence, reading ability and handedness using, respectively, Weschler Abbreviated Scale of Intelligence (WASI), Test of Word Reading Efficiency (TOWRE), and Annett Handedness Questionnaire. Any subjects with a full scale IQ <70 were excluded.

#### Non-ADHD Control Group

Letters detailing the study were sent to approximately 600 families of children in primary and secondary schools in the Nottinghamshire region. From an initial sample who volunteered to take part, we selected a group of right-handed controls matched for age (±6 months), sex, and parental socio-economic status (SES) to a member of the ADHD group. Parental SES was assessed using the eight groups identified in the ONS SES manual (ONS, v 1.1, 2004), combined to form four subgroups (Table 1) to enable accurate demographic matching. Parents completed the same battery of questionnaires used in the ADHD assessment, including a shortened version of the DAWBA. Potential participants with attention scores >1 SD above the mean on the SDQ or Conners (n = 6), or with known or suspected autistic or other major psychiatric disorders (assessed using the SCQ and DAWBA) or a FSIQ <70 were excluded from the study.

Participants undertook additional tasks (not reported in this paper) on two separate days as part of the fMRI/EEG study, in which testing was conducted on and off stimulant medication, the order of which was counterbalanced across subjects. Therefore, approximately half on the structural scans for the ADHD group were taken while the subjects were on medication (n = 13), while the remainder (n = 12) were scanned following a ∼36-hour stimulant-medication withdrawal. No effects of medication status at the time of scanning on brain structure were predicted for the ADHD group; controls were never medicated.

### MRI Acquisition and Analysis

T1-weighted (T1W) brain images in the sagittal plane were obtained with a Philips Achieva 1.5-T MRI scanner with an eight-channel SENSE head coil using a 3D TFE sequence with the following parameters: 160 contiguous slices; TR/TE 9.9/3.7 ms; matrix size, 256 × 256; voxel size, 1 × 1 × 1 mm. Head movement was minimized by the use of foam pads placed within the head coil.

The acquired images were processed using a pipeline adapted from the Montreal Neurological Institute (MNI) approach.^29^ First, we corrected nonuniformity in the intensity of T1-weighted (T1W) images using the N3 algorithm.^30^ After nonuniformity correction, the T1W images were linearly and nonlinearly registered onto the standard stereotaxic space. The template brain used here is the average brain computed from a population (SYS333) comprising 183 female adolescents (age [mean ± SD], 183 ± 24 months; FSIQ, 105 ± 12) and 150 male adolescents (age, 183 ± 22 months; FSIQ, 105 ± 13).^31^ The SYS333 template is aligned with the MNI-305 template,^32^ which is aligned with the Talairach and Tournoux atlas.^33^ The tissues were then classified into GM, white matter (WM), and cerebrospinal fluid (CSF) using the fully automated landmark-based MNI classifier.^34^ By back-projecting these tissue maps and a standard-space lobar atlas onto the native space of the original MR scans, we could quantify the overall amount of GM, WM, and CSF for each lobe in each subject. This provided a fully automated estimate of lobar volumes of GM and WM. A brain mask of the SYS333 template, obtained by manually adjusting the automatic extraction produced by the BET algorithm (FSL software), was also nonlinearly backprojected onto the native space of each scan to provide an estimate of brain size for each subject.

Estimates of cortical thickness were obtained using FreeSurfer.^35^ For each subject, GM, WM, and noncortical structures were segmented and a triangular mesh was used to measure the distance from the pial surface to the GM/WM boundary for each hemisphere.^36^ For both the FreeSurfer and the volumetry pipeline, a quality control inspection assessed for gross structural abnormalities, accuracy of registration, and presence of artifacts.

All statistical analyses were conducted using SPSS v.16 (SPSS Inc., Chicago, IL). Volumes of WM and GM and mean cortical thickness were computed for each lobe and combined across the left and right hemispheres. To ascertain the presence of group differences in frontal cortex, particularly inferior frontal gyrus (IFG), we also tested differences between the two groups in cortical thickness in 13 frontal regions segmented by the FreeSurfer. If the data violated the assumptions of sphericity, Greenhouse-Geisser adjusted degrees of freedom were used and a corrected p value was reported (p_GG_). All reported analyses include data from all eligible participants. When the analyses were repeated omitting the one female ADHD–control pair, the pattern of results remained the same.

## Results

### Participant Characteristics

There were no significant differences in age, sex, or socioeconomic status (SES) between the two groups (Table 1). Because GM and WM volume changes during development are well established, and as pairwise matching was not used in order to maximise subject numbers, AGE was used as a covariate (linear and quadratic) in all analyses. Only one analysis was improved by the inclusion of a quadratic term and in all analyses, the findings remained robust when this term was included. Controls (CTRL) had higher mean Full Scale Intelligence (FSIQ) than children with ADHD (105, SD = 15 vs. 90, SD = 12 respectively); *t* (50) = 4.27, p = .0003 and FSIQ was also used as a covariate in all subsequent analyses.

### Brain Volume

An analysis of covariance (ANCOVA) using the between subjects factor GROUP (ADHD vs. CTRL) and the covariates AGE and full-scale IQ (FSIQ), returned significant main effects of GROUP [F (1, 48) = 9.61, p = .003] and AGE [F (1, 48) = 15.52, p = .0003] but not FSIQ [F<1]. Participants with ADHD had smaller total brain volume than CONTROLS (ADHD = 1273.42 cm^3^, SD = 118.19; CTRL = 1387.89 cm^3^ SD = 112.12). Age was a significant predictor of brain volume, with larger brains in the older children. Including a quadratic term for age did not improve the fit of the model.

### Gray Matter

Absolute GM volumes for each lobe were entered into a multivariate analysis of covariance. There were significant main effects of AGE, F (4, 45) = 6.03, p = .001 and GROUP, F (4, 45) = 5.71, p = .001. Younger children had lower GM volume than older children, and children with ADHD had less GM than CONTROLS. Univariate analysis showed smaller GM volumes in all four lobes in ADHD, as compared with CONTROLS: Frontal [F (1, 48) = 17.78, p = .0001]; Parietal [F (1, 48) = 14.90, p = .0003]; Temporal [F (1, 48) = 8.01, p = .007] and Occipital [F (1, 48) = 10.60, p = .002] (Table 2). Although this GM difference is partially accounted for by the reduction in brain volume in the ADHD group, when brain volume was entered as a covariate and total GM volume for the four lobes was entered as a dependent variable, ADHD participants still had smaller (relative) GM volumes [F (4, 44) = 2.91, p = .032]. Using univariate statistics, only parietal [F (1, 47) = 4.76, p = .034] (ADHD = 117.06 cm^3^, SD = 9.25; CTRL = 123.57 cm^3^, SD = 10.08) and frontal lobe [F (1, 47) = 6.67, p = .0013] (ADHD = 240.42 cm^3^, SD = 15.90; CTRL = 253.08 cm^3^; SD = 15.75) relative GM volumes were significantly different between the two groups. The same was also true when total surface (cortical) area was entered as a covariate (instead of brain volume). Overall, total relative GM volume is significantly lower in ADHD children, as compared with CONTROLS, after covarying for surface area (or brain volume), surface ratio, and mean thickness.

### White Matter

When the analysis was repeated using WM volume, there was no main effect of GROUP (*F*<1). The smaller brain volume in the ADHD cases appears to be driven by lower GM volume in all lobes.

### Cortical Thickness

**FIGURE 1:**
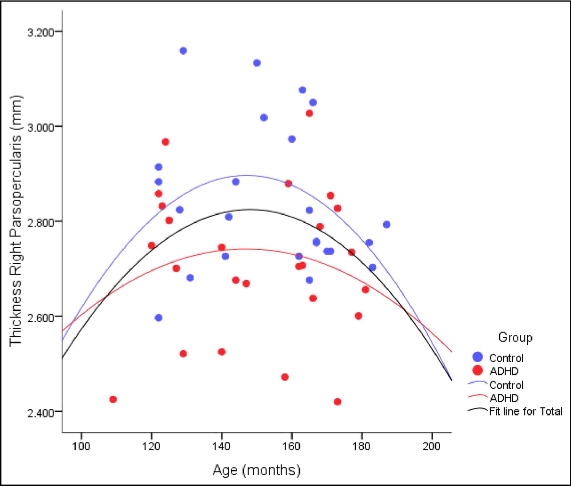
Developmental trajectory of cortical thickness of the right pars opercularis in ADHD and control groups (quadratic fit). ADHD = attention-deficit/hyperactivity disorder.

Cortical thickness measures derived from FreeSurfer were analysed in a series of ANCOVAs. Data from three control subjects were not included in this analysis because of poor registration of their scans. To test our *a priori* hypothesis that children with ADHD would show abnormalities in the Inferior Frontal Gyrus, specifically, the pars opercularis, a repeated-measures ANCOVA was conducted with HEMISPHERE as a within-subjects factor and GROUP as a between-subjects factor, using the covariates Age and FSIQ. Hemisphere was included in this analysis owing to the putative lateralised role of the right pars opercularis in inhibitory control. The ANCOVA returned a significant main effect of GROUP, F (1, 45) = 6.00, p = .018; children with ADHD had significantly thinner cortex in the pars opercularis than CONTROLS (2.71 mm, SD = .14 vs 2.81 mm, SD = .14 respectively). There was no significant difference in the thickness of the pars opercularis between the left and right hemisphere, as evidenced by the nonsignificant GROUP × HEMISPHERE interaction (F = 1.1) but univariate analysis conducted on each hemisphere separately showed a significant main effect of GROUP in the right hemisphere, F (1, 45) = 5.79, p = .02,and a trend in the left hemisphere, F (1, 45) = 3.30, p = .076. Including a quadratic term for age improved the fit of the model, whereby the left pars opercularis had a nonsignificant positive value and the right pars opercularis had an inverted U function (significant quadratic effect) of age, peaking at around 148 months (Figure 1).

**FIGURE 2:**
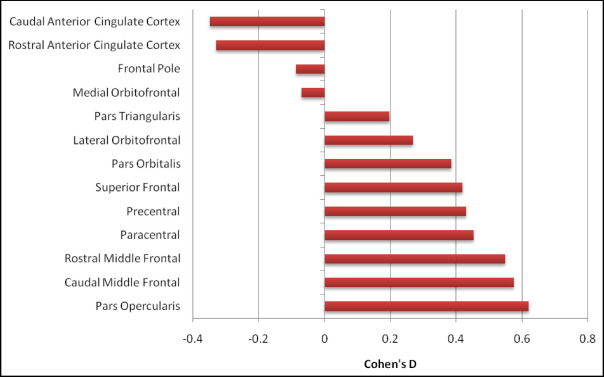
Ranked effect sizes for mean differences in cortical thickness for frontal regions in ADHD and control groups after controlling for FSIQ and Age. Positive effect sizes indicate thinner cortex in ADHD and negative effects sizes mean thicker cortex in ADHD. ADHD = attention-deficit/hyperactivity disorder; FSIQ = full-scale intelligence quotient.

Mean cortical thickness across the 13 frontal cortical regions (caudal anterior cingulate, caudal middle frontal, frontal pole, lateral orbitofrontal, medial orbitofrontal, paracentral, pars opercularis, pars orbitalis, pars triangularis, precentral, rostral anterior cingulate, rostral middle frontal, and superior frontal) were then entered into a 2 (GROUP) × 13 (REGION) × 2 (HEMISPHERE) repeated-measures ANCOVA using the covariates AGE, and FSIQ and returned a significant REGION × GROUP interaction, *F* (5.82, 262.04) = 2.49, p_(GG)_ = .024. In a planned comparison, the thickness of the pars opercularis was compared with the mean thickness of all the other frontal areas and a significant effect was found, *F* (1, 45) = 4.58, p = .038. Children with ADHD had thinner cortex in the pars opercularis relative to CONTROLS in both hemispheres. The effect sizes for each of the 13 regions are presented in Figure 2. As predicted, the largest effect was for pars opercularis (Cohen's D = 0.6).

A vertex-based analysis using the between-subjects factor GROUP failed to reveal any significance difference in cortical thickness for any voxels in any of the lobes after correcting for multiple comparisons across the entire cerebral cortex.

### Behavioral Correlate

On a Go/No-go task, inhibition rates were significantly lower in children with ADHD than controls (Table 1). Pearson correlations were computed separately for each group to determine whether there was an association between mean cortical thickness of the pars opercularis and discriminability (measured using D prime scores) on the Go/No-go task from the 39 children with available D prime data. In the children with ADHD, task performance did not correlate with thickness of the pars opercularis (r = .201, n = 21, p = .383), whereas in the control group, there was a nonsignificant trend (r = .44, n = 18, p = .071).

## Discussion

Consistent with previous studies,^16,17^ the mean total brain volume in children with ADHD was less than that of the typically developing controls. In addition, GM (but not WM) volume was lower in all four lobes. Smaller global GM volumes have been observed in other studies,^17^ particularly in frontal areas, as reviewed elsewhere.^19,37^

The global lower GM volume in the ADHD group might be explained using a model of delayed brain maturation.^38^ In both typically and atypically developing children, GM and WM development follows a similar trajectory, with GM increases in childhood followed by a reduction in adolescence. Shaw et al.^38^ measured the peak age at which cortical thickness occurred as a proxy for cortical maturation. Peak cortical thickness in children with ADHD was found to lag by approximately 4 years relative to controls, with the largest difference (∼5 years) observed in prefrontal cortex, suggestive of a maturational lag. However, cortical thinning in networks thought to subserve attention has also been found in adults with ADHD,^39^ indicating that developmental structural anomalies in ADHD might persist into adult life rather than normalise with age. In support of this view, Castellanos et al.^16^ used a longitudinal design and found that volume abnormalities present in children with ADHD continued into adulthood. Although the neurobiological underpinnings of these group differences in GM are unknown, they could be related, for example, to fewer synapses and/or reduced dendritic branching in children with ADHD, with corresponding decreases in cerebral metabolism accompanied by a reduction in the numbers of glial cells.^40^ Whereas widespread reductions in GM appear robust and are of interest in their own right, we were particularly interested in determining whether cortical abnormalities in specific structures associated with control of inhibition might also be present in ADHD. We began with an *a priori* interest in the frontal lobe, specifically, the pars opercularis, because of its putative role in inhibitory control.^9,10,22^ As hypothesized, the cortex was thinner throughout the frontal lobe in the children with ADHD, but was significantly thinner in the pars opercularis, which also had the greatest effect size. Of note, and in accord with its proposed role in inhibitory function, not only did the children with ADHD show structural differences in the pars opercularis, but their performance on a Go/no-go task was also significantly poorer (d′ scores in Table 1). Furthermore, although there was a nonsignificant trend for performance to be positively correlated with cortical thickness in the pars opercularis in the control group, no such trend was observed in the ADHD group. This finding is not wholly unexpected, given that participants were selected on the basis of having good (control participants) or poor (ADHD participants) inhibitory control. Thus, the weak or absent association between structure and behaviour in each group is likely to reflect homogeneity within groups. Similar findings were evidenced in a study by Durston et al.^13^ Using fMRI, children and adolescents with ADHD, their unaffected siblings and controls undertook a Go/No-go task. Correlations between right IFG and performance were evident in controls and unaffected siblings only and not in participants with ADHD. In the current study, thinner cortex was present in both the left and right hemisphere in the children with ADHD. Our finding of a larger effect in right IFG but no significant hemispheric difference, is consistent with prior functional imaging studies of Go/No-go tasks, which more commonly show engagement of right IFG^14^ but in some instances report engagement of bilateral IFG.^41^

The strengths of this study include a well-defined clinical sample, the inclusion of only combined type ADHD and careful matching of controls. There are, however, also some limitations. Although the sample size is larger than that of many similar studies, it lacked the statistical power required to explore multiple *a priori* regions or to detect small effects post hoc.^16,38^ Although the groups were carefully matched in terms of demographic factors, they were not matched for IQ. However, as participants came from similar areas and backgrounds, the lower IQ scores in the ADHD group are likely to be a consequence of the disorder rather than other factors such as social disadvantage, and thus reflect “typical” ADHD. Indeed, attention and learning problems are highly interrelated and typically coexist.^42^ As expected,^21^ comorbidity—particularly behavioral disorders—was present in most participants with ADHD, all of whom were taking long-term stimulant medication. Nonetheless, when the analysis was repeated including only those participants with externalising disorders (ODD/CD), the results remained robust despite the reduction in sample size. This finding is in keeping with other studies in which comorbid behavioral disorders such as oppositional defiant disorder and conduct disorder have evidenced relatively little additional effect on brain structure in ADHD.^15^

With respect to medication, in one of the largest morphologic studies to date,^16^ no significant differences were found between medicated and treatment-naive subjects, suggesting that medication has little effect on brain structure. In contrast, Semrud-Clikeman et al.^43^ found reductions in right anterior cingulate cortex volume in treatment-naive relative to medicated ADHDs and controls, raising the intriguing possibility that medication may “normalize” deficient structures by strengthening connections within the structure in much the same way that synaptic plasticity may increase the size of local structures through demand.^44^ Further support for this notion is provided in a recent study by Shaw et al.,^45^ who found more rapid cortical thinning in excess of age-appropriate rates in children with ADHD not taking stimulant medication. If so, then the morphological reductions observed in our ADHD participants may be an underestimate of the true effects of ADHD rather than a consequence of stimulant medication.

Our findings demonstrate that an ADHD combined type subgroup, with clinical features including impulsivity/hyperactivity have both a generalized deficit in gray matter compared with healthy controls, but there is some evidence of nonuniformity, with the deficits being most marked in IFG. Future work using a single heterogeneous group with a spectrum of severity of impulsivity deficits would enable us to test for the anatomical correlates of impulsivity in ADHD.

## Figures and Tables

**TABLE 1:** Clinical and Demographic Characteristics of Subjects

	Group		*p* Value
ADHD N = 25	Control N = 24
Gender	M=24, F=1	M=23, F=1	n/s
Age (y)	12.48 (1.86)	12.82 (1.69)	.52
Weight (kg)	42.84 (15.30)	48.38 (11.87)	.17
Duration of stimulant medication (mo)	38.1 (23.7)	n/a	n/a
D′^a^	1.16	1.68	.012
FSIQ	89.84 (11.65)	104.67 (14.74)	.0003
Total digit span scaled	7.72 (2.76)	9.75 (3.05)	.018
TOWRE: Total Score	89.56 (22.34)	98.63 (13.99)	.097
Conners Parent DSM total	81.40 (7.50)	44.79 (6.17)	4.21E-23
SES classification (n)			.89
Higher professional	1	1	
Lower professional	5	5	
Self-employed	1	1	
Manual/unemployed	18	17	
Co-morbid diagnoses			
ODD	11	0	n/a
CD	7	0	n/a
DCD	1	0	n/a
RD	1	0	n/a
GAD	3	0	n/a
Depression^b^	1	0	n/a
Specific phobia	3	0	n/a

*Note:* Some subjects had more than one comorbidity. ADHD = attention-deficit/hyperactivity disorder; CD = conduct disorder; DCD = developmental coordination disorder; DSM = Diagnostic and Statistical Manual of Mental Disorders; FSIQ = full-scale intelligence quotient; GAD = generalized anxiety disorder; ODD = oppositional defiant disorder; RD = reading disorder; SES = socioeconomic status; TOWRE = Test of Word Reading Efficiency.

a D prime scores are taken from a Go/No-go task using 24 matched pairs (ADHD participants off medication; Cohen's Dz effect size = 0.76).

b Mild depressive episode.

**TABLE 2:** Absolute Gray Matter Volumes by Lobe and Group

	Group			
ADHD		Control	
Mean (cm^3^)	SD	Mean (cm^3^)	SD
Total frontal GM absolute	232.87	21.67	260.07	21.55
Total parietal GM absolute	113.23	12.09	127.16	12.01
Total temporal GM absolute	161.03	15.91	174.43	15.82
Total occipital GM absolute	67.30	8.76	75.79	8.71

*Note:* ADHD = attention-deficit/hyperactivity disorder; GM = gray matter.
